# Differences in Gut Microbiome Composition between Senior Orienteering Athletes and Community-Dwelling Older Adults

**DOI:** 10.3390/nu12092610

**Published:** 2020-08-27

**Authors:** Frida Fart, Sukithar Kochappi Rajan, Rebecca Wall, Ignacio Rangel, John Peter Ganda-Mall, Lina Tingö, Robert J. Brummer, Dirk Repsilber, Ida Schoultz, Carl Mårten Lindqvist

**Affiliations:** 1 School of Medical Sciences, Faculty of Medicine and Health, Örebro University, 702 81 Örebro, Sweden; frida.fart@oru.se (F.F.); sukithar.rajan@oru.se (S.K.R.); rebecca.wall@oru.se (R.W.); ignacio.rangel@oru.se (I.R.); john-peter.ganda-mall@oru.se (J.P.G.-M.); lina.tingo@oru.se (L.T.); robert.brummer@oru.se (R.J.B.); dirk.repsilber@oru.se (D.R.); ida.schoultz@oru.se (I.S.); 2 Laboratory of Translational Mucosal Immunology, Digestive Diseases Research Unit, Vall d’Hebron Institut de Recerca, Hospital Universitari Vall d’Hebron, 08035 Barcelona, Spain

**Keywords:** gut microbiota, metagenomics, aged, *Faecalibacterium prausnitzii*, orienteering

## Abstract

Background: Gastrointestinal (GI) health is an important aspect of general health. Gastrointestinal symptoms are of specific importance for the elderly, an increasing group globally. Hence, promoting the elderly’s health and especially gastrointestinal health is important. Gut microbiota can influence gastrointestinal health by modulation of the immune system and the gut–brain axis. Diverse gut microbiota have been shown to be beneficial; however, for the elderly, the gut microbiota is often less diverse. Nutrition and physical activity, in particular, are two components that have been suggested to influence composition or diversity. Materials and Methods: In this study, we compared gut microbiota between two groups of elderly individuals: community-dwelling older adults and physically active senior orienteering athletes, where the latter group has less gastrointestinal symptoms and a reported better well-being. With this approach, we explored if certain gut microbiota were related to healthy ageing. The participant data and faecal samples were collected from these two groups and the microbiota was whole-genome sequenced and taxonomically classified with MetaPhlAn. Results: The physically active senior orienteers had a more homogeneous microbiota within the group and a higher abundance of *Faecalibacterium prausnitzii* compared to the community-dwelling older adults. *Faecalibacterium prausnitzii* has previously shown to have beneficial properties. Senior orienteers also had a lower abundance of *Parasutterella excrementihominis* and *Bilophila* unclassified, which have been associated with impaired GI health. We could not observe any difference between the groups in terms of Shannon diversity index. Interestingly, a subgroup of community-dwelling older adults showed an atypical microbiota profile as well as the parameters for gastrointestinal symptoms and well-being closer to senior orienteers. Conclusions: Our results suggest specific composition characteristics of healthy microbiota in the elderly, and show that certain components of nutrition as well as psychological distress are not as tightly connected with composition or diversity variation in faecal microbiota samples.

## 1. Introduction

During the last decade, longevity has increased among the elderly population, resulting in a global ageing phenomenon that is having a major impact on healthcare systems worldwide. This has led to an increased awareness of the importance of promoting healthy ageing and quality of life throughout an individual’s lifespan. To promote and initiate healthy ageing, it is important to understand and reveal the underlying mechanisms.

**Figure 1:**
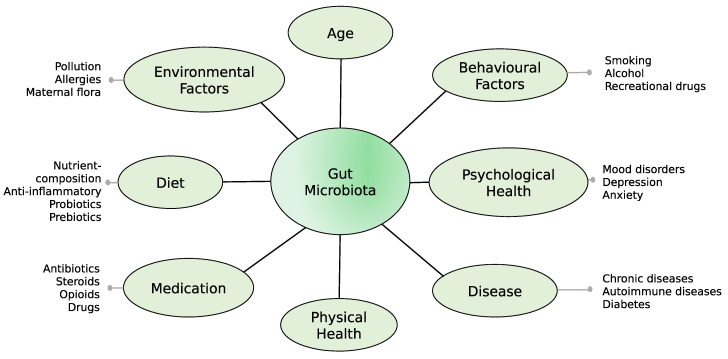
Factors affecting the composition of gut microbiota.

The gastrointestinal (GI) tract is an essential part of the human body and physiological system through which health and well-being might be promoted [1]. A well-functioning GI tract has previously been identified as crucial for subjective health and well-being among older adults [2]. GI symptoms are common among community-dwelling older adults (i.e., older adults residing in their own household) in Sweden, and as many as 65% experience one or several gut symptoms that correlate with increased psychological distress, including anxiety and depression [3]. On the contrary, physically active seniors engaged in orienteering (a sport involving finding specific locations using a map and a compass) have previously been identified as a potential model of healthy ageing [4], as they display the three main components of successful ageing—physical endurance, cognitive skills, and social interaction [5]. Indeed, our previous data show fewer GI symptoms among senior orienteers and a better overall health compared to community-dwelling older adults [4,6]. This indicates that gut health may reveal important factors of well-being in the elderly, especially its association with various factors that are known to influence gut microbiota during the entire lifespan. The microbial composition of an individual depends on factors such as age, diet, geography, environmental exposure, and many others, as shown in Figure 1 [7,8,9,10].

The human GI tract is a complex ecosystem where the gut microbiome interplays with host cells and dietary-derived components, both of which have been implicated in playing a major role in health and disease [1]. A diverse gut microbiome has been related to several essential mechanisms for both a well-functioning GI tract as well as well-being, including modulating the immune system, maintaining an intact intestinal barrier, and being a part of the regulation of the gut-brain axis [11,12], where a decreased diversity has been linked to both GI and psychiatric disorders [13]. Ageing has been associated with a loss of diversity of the gut microbiome; specifically, bacteria belonging to the phyla *Firmicutes* and *Actinobacteria* decrease, whereas *Proteobacteria* increase in abundance [14]. These changes could be due to nutritional deficiencies such as lower intakes of specific nutrients, e.g., dietary fibres and proteins, that are important for maintaining the immune and GI functions [15]. Recent evidence further indicates that physical activity, independent of diet, could induce positive alterations of the gut microbiome composition [16,17]. However, the relationship between physical activity and gut microbiota across the life course has not been entirely elucidated. It is also less known to what extent microbiome composition and diversity are influenced by certain factors when other factors change at the same time, especially in a diverse population such as the elderly. For example, it is still not clear which specific influence could be attributed to nutrition components or psychological factors such as distress or anxiety.

In the present study, we investigated the gut microbiota profile in senior orienteering athletes, as a proposed model of healthy ageing, in relation to GI symptoms and macronutrient intake and compared it to the gut microbiota composition of community-dwelling older adults, representing the general older adult population, to identify possible patterns specifically related to healthy ageing.

## 2. Materials and Methods

### 2.1. Study Participants, Data Collection, and Ethics

Samples were available from two previously established cohorts: community-dwelling older adults (hereafter referred to as older adults), representing a cross-section of the general older adult population [3,18] (*n* = 70) and physically active senior orienteers (hereafter referred to as senior orienteers) as a model of healthy ageing [6] (*n* = 28). All participants were ≥65 years of age; the inclusion and exclusion criteria are presented in Table 1. The study received approval from the Regional Ethics Board in Uppsala, Sweden (dnr: 2012/309, 2013/037, 2015/357) and was conducted in accordance with the Declaration of Helsinki.

### 2.2. Gastrointestinal Symptoms, Psychological Distress, and Physical Activity

Data regarding GI symptoms, psychological distress, and physical activity were available from the two previously established cohorts for all orienteers and a subset of older adults (*n* = 54) [3,6]. GI symptoms and psychological distress were assessed through the following validated questionnaires: the Gastrointestinal Symptom Rating Scale (GSRS) [19] and the Hospital Anxiety and Depression Scale (HADS) [20]. Briefly, the GSRS comprises 15 questions assessing five GI symptoms (i.e., reflux, abdominal pain, dyspepsia, diarrhoea, and constipation) that are scored from 1 to 7 depending on their severity. A total score is then calculated as the average from the five symptom scores. The HADS includes 14 questions and is divided into two subscales assessing anxiety and depression (7 questions/scale) together giving an estimation of psychological distress. The Frändin–Grimby Activity Scale (FGAS) [21], a 6-point scale with fixed response alternatives, was used to assess the level of physical activity.

### 2.3. Macronutrient Intake

The nutrient intake was estimated by a validated semi-quantitative Food Frequency Questionnaire (FFQ) [22] asking for dietary intake during the past year. The questionnaire has previously been described and used in an elderly population [3]. Raw data were available from the previously established cohorts [3,18] and were further analysed according to a standard procedure to assess the following macronutrients: fibre, protein, saturated fat, unsaturated fat, and carbohydrates as well as estimated added sugar. Briefly, participants estimated their intake of 66 food items from 0–8 (0 = never, 8 = 4 or more times a day). To facilitate inter-individual comparisons, the intake per day was expressed as energy percentage (E%) and the intake of fibre was expressed as gram per megajoule (MJ) energy intake.

### 2.4. Medications

Medications were self-reported and grouped according to the Anatomical Therapeutic Chemical (ATC) classification system, controlled by the WHO’s Collaborating Centre for Drug Statistics Methodology, by a physician (author F.F.), using a national tool [23].

### 2.5. Next-Generation Sequencing for Determination of the Microbiota Composition

Stool samples were collected according to standard operating procedures [18] and were analysed using next-generation sequencing (NGS) for assessment of the faecal microbial composition [24]. Total DNA was extracted from faecal samples using a QIAmp DNA stool mini kit according to the manufacturer’s instructions (Qiagen, Hilden, Germany), coupled with an initial bead-beating step. The total microbial content was further assessed through whole-genome sequencing (WGS) at SciLifeLab, (Stockholm, Sweden) using an Illumina HiSeq 2500 device (Illumina, San Diego, CA, USA) with four samples per lane, yielding approximately 50 million read pairs per sample. Whole-genome sequences were taxonomically classified using MetaPhlAn v2.0 (Huttenhower Lab, Boston, MA, USA) [25] at default settings. Relative abundances for the taxomic levels of genera and species were extracted from the output of MetaPhlAn and further analysed in R (3.6.1, R Core Team, New Zealand) [26].

### 2.6. Data Analysis

Continuous demographic data were analysed using the Mann–Whitney U test, and categorical demographic data were analysed using the chi-square test. Relative abundances for microbiota at genus and species level were calculated and considered for further analysis. Welch’s two-sample *t*-tests followed by the Benjamini–Hochberg procedure for multiple testing correction were used to assess the difference in bacterial abundance between the two groups [27]. Top genera, differentially occurring in orienteers and general elderly, were selected based on a false discovery rate (FDR) less than 5%. Given our abundance data and group sizes (orienteers, older adults), we estimated to be able to detect a 20% difference in abundance, with 80% power and 5% significance level. Species representing the top predicted genera were considered for further downstream statistical analysis. To estimate if a difference was consistent after the effect of the covariate was taken into account, we fitted a zero-inflated negative binomial (ZINB) regression model with each covariate as an explanatory variable [28,29,30]. The resulting residuals were considered as corrected bacterial abundances with the effect of the covariate removed. The differences of these corrected bacterial abundances between groups were tested using a ZINB model and ANOVA type III sums of squares test for the bacterial abundances. The relative importance of all covariates was assessed by performing a model comprising all covariates using likelihood-ratio chi-square statistics [31].

All plots were produced in R (version 3.6.1, R Core Team, New Zealand) [32] using either the base graphics package or ggplot2 version 3.2.1 [33]. Boxplots were produced with the graphics package using the notch option, where box encapsulates the first to third quantiles and whiskers are the minimum of 1.5 interquartile range (IQR) from the box or the min/max value. Boxplot notches visualise a non-parametric estimation of the 95% confidence interval of the median calculated as +/-1.58 IQR/√n [34]. ANOVA with type III sums of squares analysis was performed as implemented in the car package v 3.0-3. Zero-inflated negative binomial regression was performed using the function zeroinfl within the pscl package v 1.5.5 [35]. FDR values were estimated using the package multtest, following the approach adopted by Benjamini and Hochberg [36]. Bray-Curtis distances and Shannon diversity index were calculated from species abundance profiles using the vegan (v2.5-6) package [37]. PCoA analyses were performed with the R package labdsv [38]. Participants outside the 95% confidence area formed the subset atypical older adults. Student’s *t*-test was performed to compare average values between senior orienteers, typical older adults, and the subset of atypical older adults for each covariate. Taxonomy prediction and statistical analysis were automated using in-house scripts written in Bash and R (Figure S1) [26].

## 3. Results

### 3.1. Demographic Data

All demographic data are presented in Table 2. The degree of anxiety (*p* = 0.006) and depression (*p* = 0.002) were significantly higher among older adults compared to senior orienteers, whereas physical activity was higher among senior orienteers (*p* < 0.001).

### 3.2. Microbiota Composition

**Figure 2:**
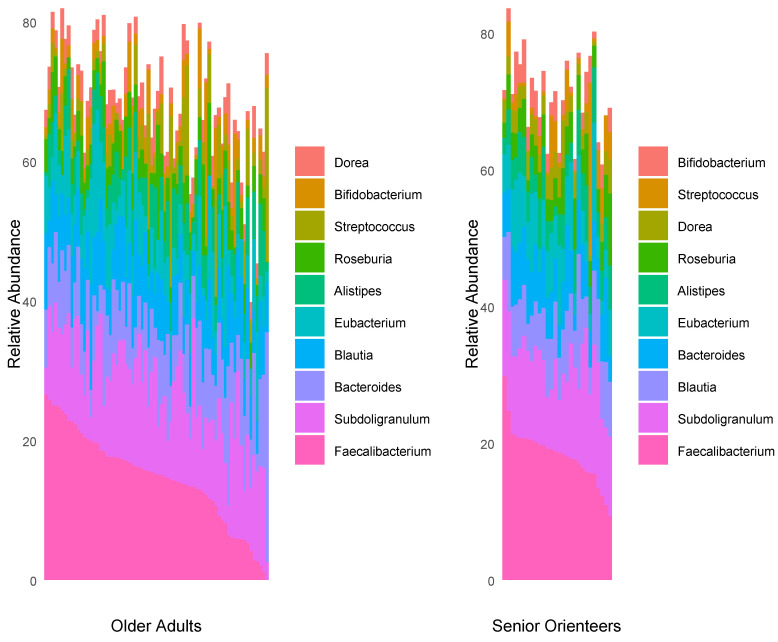
Relative abundance of the 10 most abundant genera across 98 samples.

**Figure 3:**
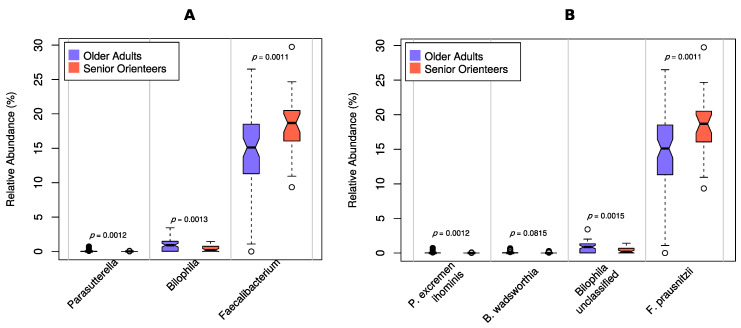
Relative abundance of significantly different genera and selected species stratified for group (senior orienteers compared to older adults). Cut-off for significance was set at false discovery rate (FDR) <5%. Descriptive *p*-values for each comparison are shown. (**A**) Genera; (**B**) Species.

Faecal microbiota profiles of the two established cohorts of older individuals (senior orienteers and older adults) were analysed on both genera and species levels from shotgun metagenomic sequences. *Faecalibacterium* was on average the most prominent genus and a total of 111 genera were found in at least one sample (Figure 2, Supplementary Table S1). Three of these genera showed significantly different proportions between senior orienteers and older adults (Figure 3A). These three genera are represented by four species that were used for further analysis. Of these four species, *Faecalibacterium prausnitzii* and *Bilophila* unclassified were the most abundant (Figure 3B).

**Figure 4:**
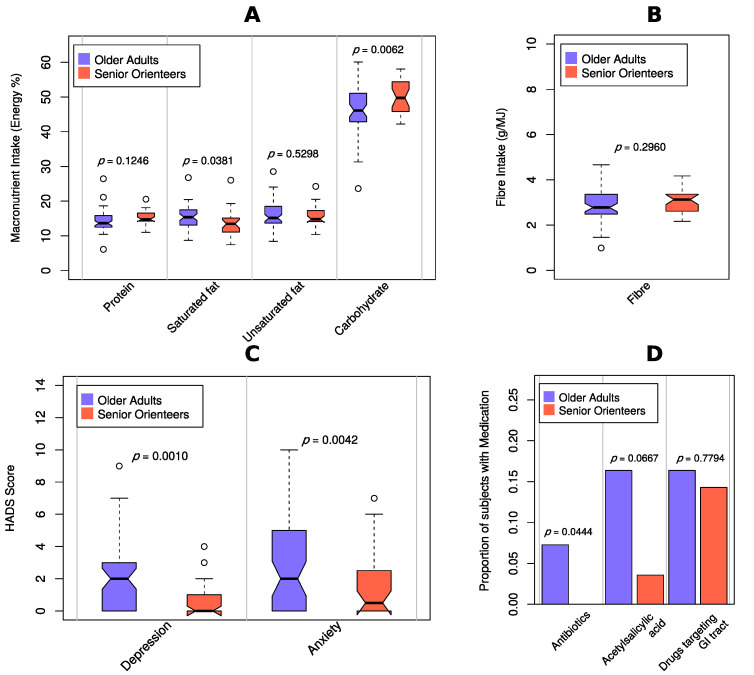
Comparison of covariates. Boxplots of covariates stratified for older adults and senior orienteers, including descriptive *p*-values from Welch’s *t*-test. (**A**) Macronutrients measured by energy percentage (E%). (**B**) Fibre measured by grams per megajoule (MJ). (**C**) Hospital Anxiety and Depression Scale (HADS) score. (**D**) Bar plot for medication covariates for older adults and senior orienteers, including descriptive *p*-values from chi-square test.

**Figure 5:**
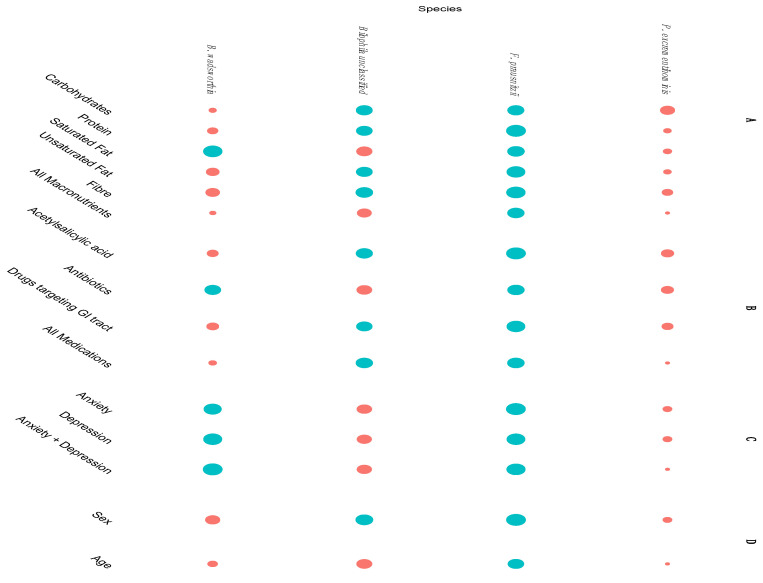
Significance of difference between older adults and senior orienteers after correction for macronutrients, psychological distress, and medication variables. Corrected bacterial composition values were compared between groups for each species and false discovery rates (FDRs) calculated. The dots represent negative log10 *p*-values belonging to respective species, where blue denotes significance and red denotes non-significance, with a significance threshold at FDR <5%. A Results for models with a single macronutrient variable and with all macronutrient variables in a multi-variable model. B Results for models with single medication variables and with all variables in a multi-variable model. C Results for models regarding anxiety and depression separately with single Hospital Anxiety and Depression Scale (HADS) variables and with both HADS variables in a multi-variable model. D Results for models with sex and age.

**Figure 6:**
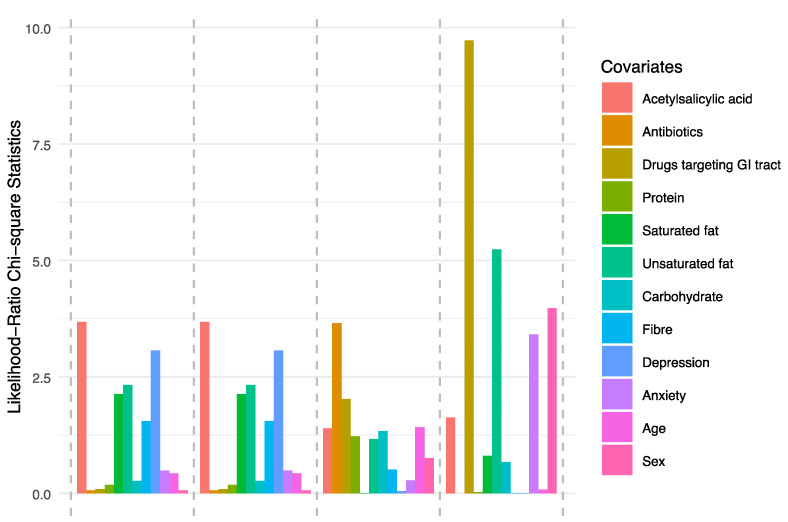
Assessment of relative importance of all covariates. A complete model comprising all covariates for assessing variable importance. The relative importance of each covariate was measured as likelihood-ratio chi-square statistics.

To investigate whether differences of microbiota composition were due to confounding factors, twelve covariates were included in the analyses, i.e., five macronutrients (carbohydrates, protein, unsaturated fat, saturated fat, and fibre), two parameters assessing psychological distress (anxiety and depression), three parameters assessing medicines associated with dynamic changes in the microbiota (antibiotics during the previous six months, acetylsalicylic acid, and any medicine affecting the GI tract), sex, and age (Figure 4, Figure 5 and Figure 6). Several covariates were significantly different between the groups. The senior orienteers had a significantly higher intake of carbohydrates and a lower intake of saturated fat in their diet compared to older adults (*p* = 0.006 and *p* = 0.038, nominal *p*-values). Older adults reported a higher level of depression and anxiety (Table 1). One species was significantly increased in senior orienteers after correcting for all covariates, namely *Faecalibacterium prausnitzii. Bilophila* unclassified was significantly different for 8/15 covariates or combinations of covariates, and *Bilophila wadsworthia* as significantly different for 5/15 covariates or combinations of covariates (more abundant in older adults; see Figure 5).

### 3.3. Ecological Diversity and Homogeneity

**Figure 7:**
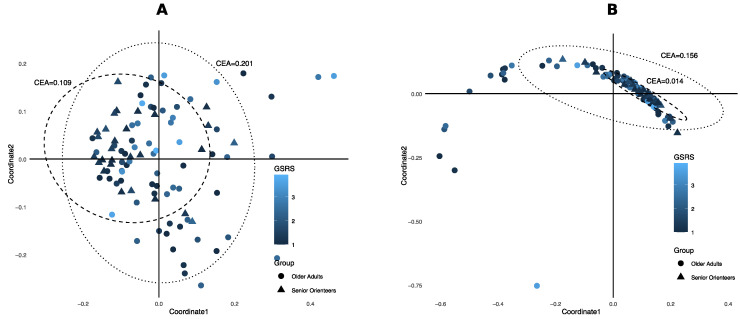
Principal coordinates analysis (PCoA) plots. Principal coordinates were estimated using Bray–Curtis distance on the predicted species. Each dot represents an individual sample, shape depicts groups, and blue scale codes for the gastrointestinal symptom scores measured with Gastrointestinal Symptom Rating Scale (GSRS) values. Dotted ellipse indicates 95% confidence region of older adults and dashed ellipse indicates 95% confidence region of senior orienteers. CEA = 95% confidence ellipse area. (**A**) PCoA using all predicted species; (**B**) PCoA using four selected species that were significantly different between older adults and senior orienteers.

**Figure 8:**
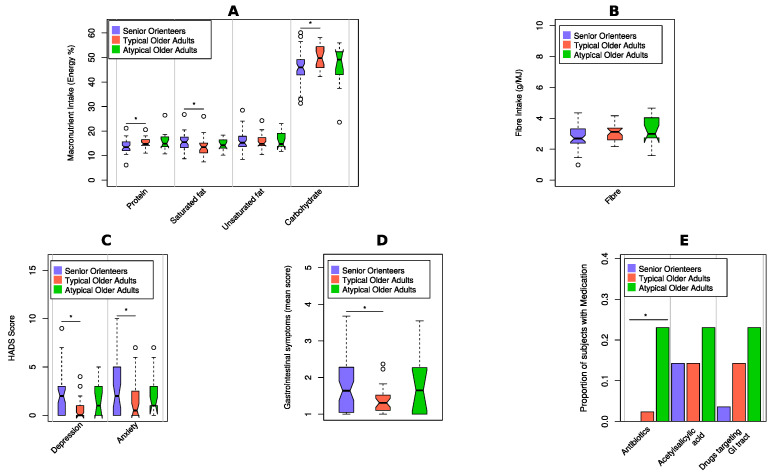
Comparison of covariates when older adults are stratified for typical and atypical. Atypical older adults are defined as samples outside of the confidence ellipse area in Figure 7. Statistically significant differences are marked with an asterisk. (**A**) Macronutrient intake measured by energy percentage (E%). (**B**) Fibre measured by grams per megajoule. (**C**) Anxiety and depression scores. (**D**) Mean score of gastrointestinal symptoms. (**E**) Representation of proportion of subjects with medications.

**Figure 9:**
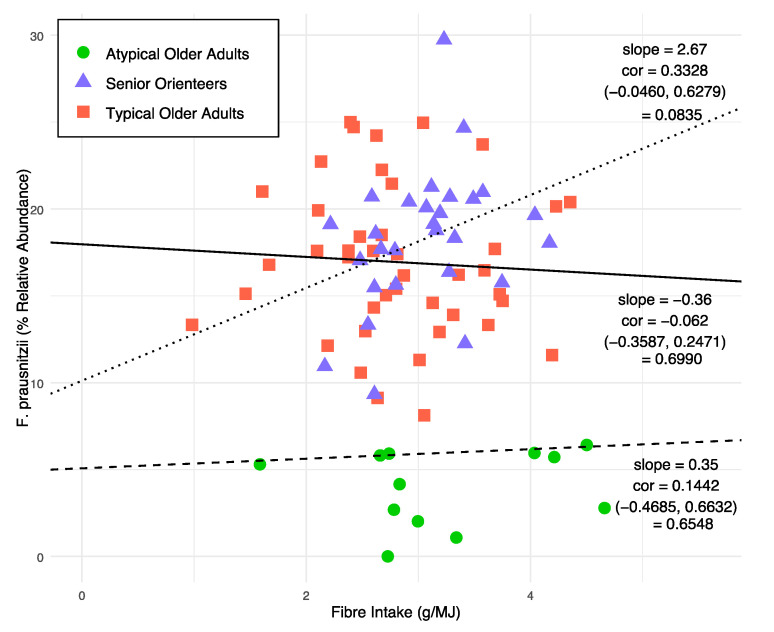
Correlation between *Faecalibacterium prausnitzii* and fibre intake. Shape depicts different groups. Dotted line, solid line, and dashed line represent regression lines for senior orienteers, typical older adults, and atypical older adults, respectively. Confidence interval (95%) values are given in brackets for respective observed correlations.

No difference in alpha diversity in terms of Shannon index was observed between the groups (Supplementary Figure S2). To estimate the beta diversity, principal coordinates analysis (PCoA) with a Bray–Curtis dissimilarity score was used. In the PCoA, the microbiota profiles of senior orienteers appear more homogenous than the profiles of older adults (95% confidence ellipse area, 0.2013 for older adults and 0.1094 for senior orienteers; see Figure 7A). When analysing only the four species that are significantly different between the groups, the homogeneity difference became even larger (0.1861 for older adults and 0.0179 for senior orienteers; see Figure 7B). Based on the PCoA with the four selected species, there appeared to be a subset of individuals, all from the older adult group, that have an atypical microbiota profile. This atypical participant group (atypical older adults, *n* = 12) was compared with orienteers (*n* = 28) and the rest of the older adults (typical older adults, *n* = 42) regarding covariates (Figure 8). Significant differences were observed only between senior orienteers and the majority group of typical older adults. Protein, saturated fat, carbohydrates, depression, anxiety, and GSRS variables showed significant differences between these two groups. Interestingly, the atypical group of older adults seems to be closer to the senior orienteering group than the typical older adults for these covariates. Confidence intervals of correlation values between *F. prausnitzii* and fibre showed a trend towards a weak correlation. A trend for a positive correlation was found between *F. prausnitzii* and fibre in the orienteering group although not significant (cor = 0.33, 95% CI = [−0.046, 0.63]). For the older adults, no such correlation could be seen (cor = −0.062, 95% CI = [−0.36, 0.25] for typical adults and cor = 0.14, 95% CI = [−0.53, 0.57] for atypical adults) (Figure 9).

## 4. Discussion

The present study focused particularly on identifying gut microbiota profiles related to healthy ageing. Collectively, the novel data of the study show that senior orienteers, used as a model of healthy ageing, display a significantly different composition of the gut microbiota, with higher levels of *F. prausnitzii*, compared to older adults. Notably, these changes were found to be persistent even after correcting for macronutrient intake, psychological distress, and medical regimen affecting the GI tract. As a higher abundance of *F. prausnitzii* is associated with good gastrointestinal health [39], this result is coherent with our previous studies of the cohorts [4,6]. In addition, we observed more homogeneous overall compositions of gut microbiota in the senior orienteer cohort.

Senior orienteering athletes have previously been identified as a potential model of healthy ageing [4], where we have previously shown that signs of depression, anxiety, and gastrointestinal discomfort are lower in this group compared to older adults [6]. In the present study, assessment of the macronutrient intake further showed that senior orienteers had a lower intake of saturated fats and a higher intake of carbohydrates compared to older adults. This result further supports our previous findings that senior orienteers display several factors associated with health. The dietary intake has previously been shown to be a major factor influencing the composition of the gut microbiota and, subsequently, the metabolic output and function of the gut microbiome [40,41,42].

While a Western-style diet, rich in saturated fat and low in fibre, gives rise to a less diverse gut microbiota with a metabolic profile likely to be detrimental to health [42,43], the addition of dietary fibres, fruits, and vegetables is able to shift the composition to a more diverse composition associated with an increase in bacterial species, including *F. prausnitzii* [44,45,46]. *F. prausnitzii*, recognized as a marker of a healthy gut [39], is a non-spore forming and strict anaerobe, placed taxonomically within *Clostridium* cluster IV [47], which is a member of the *Clostridium leptum* group [48]. It is also one of the most important members among the butyrate-producing bacteria in the human colon [49,50]. The function of *F. prausnitzii* in the gut has been associated with its high capacity to contribute to the production of the short-chain fatty acid butyrate, the main nutrient for colonocytes known to display anti-inflammatory properties [51]. A diet high in fibre has previously been associated with an increased abundance of *F. prausnitzii* [52,53]. Within the senior orienteering group, we identified a trend towards a positive correlation between intake of fibres, including dietary fibres, and relative abundance of *F. prausnitzii.* As the trend is not visible in community-dwelling older adults, this result could suggest that fibre intake is linked to higher *F. prausnitzii* abundance only in a group with a lower degree of GI problems. However, it is important to note that a limitation of the study is the assessment of macronutrient intake via an FFQ estimating intake over a year. Therefore, the result may be affected by recall bias and a dietary diary would have been an excellent complement to estimate the intake during the days of stool sampling.

Our findings further show that *F. prausnitzii* accounts for approximately 18% of the total faecal gut microbiota in senior orienteers compared to 15% among community-dwelling older adults. This is in accordance with two previous independent studies showing that 5–15% of the microbiota consists of *F. prausnitzii* [39,54]. This observation may indicate that senior orienteers have a higher production of butyrate. However, butyrate was not assessed in the present study as the level of butyrate in the luminal content does not reveal whether the elevated levels are due to the gut microbiota composition or a disturbed uptake of butyrate in the intestinal mucosa. Hence, further studies are needed to elucidate how the abundance of *F. prausnitzii* correlates to butyrate production in older adults. Moreover, the high relative abundance of *F. prausnitzii* in the present study may be due to geographical location as both elderly and adult individuals in Sweden have been found to have a high abundance of this particular species compared to microbiota profiles found in other European countries [55].

Even though a higher relative abundance of *F. prausnitzii* was observed, we did not observe an enhanced microbial diversity among senior orienteers. This is in contrast to previous findings where regular exercise and sustained levels of increased physical activity have been shown to enhance microbial diversity independent of diet [16,17,56]. Interestingly, a recent report shows that, even though regular exercise among older adults is important to maintain a stable gut microbiota, the α-diversity was not significantly different between older adults performing regular exercise compared to those who did not [57]. On a family level, a change in relative abundance of several bacterial families was observed, but not in the Ruminococcaceae family, to which *F. prausnitzii* belongs. A recent systematic review further summarizes the field and shows that higher levels of physical activity and cardiorespiratory fitness are associated with higher faecal concentration of short-chain fatty acids in adults [58]. However, it was not possible to distinguish whether short-term or medium-/long-term exercise had a more positive effect on the gut microbiota composition. It is therefore possible that orienteering among elderly may only have moderate effects on the gut microbiota. It is further important to note that the level of physical activity is self-reported and does not give an exact indication of how hard the participants exercised. In addition, the present study is limited by the low number of senior orienteers, and the absence of significant differences may reflect low statistical power rather than true negative findings. Hence, more in-depth future studies are needed to thoroughly elucidate the relationship between physical activity and gut microbiota composition in the elderly.

Moreover, a physically inactive lifestyle together with a diet high in refined carbohydrate and low in dietary fibre is associated with a depleted microbiome and the elevated risk to develop chronic diseases [59]. In our study, the bacterial species *Parasutterella excrementihominis* and *Bilophila wadsworthia* were found in a higher relative abundance in community-dwelling older adults. Although little is known regarding their function, it is intriguing to note that both species have been associated with decreased intestinal health. *Parasutterella excrementihominis* belongs to the class *Betaproteobacteria* (one of eight classes of *Proteobacteria*). The relative abundance of *Parasutterella excrementihominis* has previously been associated with different host health outcomes such as inflammatory bowel disease, irritable bowel syndrome, obesity, diabetes, and fatty liver disease [60,61,62,63]. *Bilophila* is a member of the hydrogen sulphide (H_2_S)-producing family *Desulfovibrionaceae*. *Bilophila* metabolizes sulphated compounds and produces H_2_S that can trigger inflammation, exert genotoxic and cytotoxic effects on epithelial cells, and impair intestinal barrier function [64]. Correlations of sulfidogenic bacteria to the aetiology of chronic metabolic diseases have recently been shown [65,66]. However, little is known about the genus *Bilophila*. *Bilophila wadsworthia* has been associated with a variety of human and animal infections [67,68,69,70].

Another possible environmental factor that can influence gut microbiota is medications [71]. Common drugs, including antibiotics, have been found to alter the gut microbiota composition [72]. Repeated courses of antibiotic treatment may result in the loss of microbial species that may not be restored [73]. Prescribed medication from medical records would have provided appropriate data to investigate an accurate list of medications since our data did not include dosage or common usage. However, the prescribed medications do not include over-the-counter medications, which comprise several agents affecting the gastrointestinal canal directly (such as proton-pump inhibitors, laxatives, etc.). As the differences between senior orienteers and older adults are still significant after diet and medications are taken into account, the distinctive features of the former group are further accentuated as important for the differences in microbiota.

Moreover, it is important to acknowledge that senior orienteers have a lifestyle represented not only by a high level of physical activity, but also by an active social life. Loneliness and lack of contact often increase the risk of depression and anxiety among older adults [74]. In accordance with previous data, we show that depression and anxiety are significantly lower among senior orienteering athletes compared to community-dwelling older adults. Depression and anxiety are known to be associated with an altered gut microbiota composition that is most likely due to changes in the microbiota–gut–brain axis, the bidirectional relationship between the gut microbiota and brain [75]. One of the major factors influencing this pathway is diet and, among other factors, a change in eating habits due to increased psychological distress has been proposed to contribute to the alterations of the gut microbiota associated with depression and anxiety [76]. However, the relationship between diet and depression and anxiety needs to be further investigated as the results from dietary intervention studies are contradictory and the directionality and mechanisms are currently unclear as reviewed by Bear et al. [75]. In accordance with these observations, a recent systematic review of the field shows that so far there is no consensus within human studies regarding the question about which bacterial taxa would be most relevant to depression [77].

This study focused only on a few factors that could possibly have an impact on the gut microbiome. However, there are many other environmental, behavioural, socio-economic, and health-related variables that contribute to the gut microbial composition (Figure 1). The scope of this study was to investigate the difference between senior orienteers and older adults after correcting for a variety of factors. Many of the factors that were used for correcting the microbiota composition are not independent, but are different between the two studied groups and, therefore, confounded with each other. An elaborate analysis of predicted function profiles of proteins, pathways, and metabolite levels will provide more insight into the functional aspects of healthy ageing, but remains outside the scope of this study.

In healthy adults, the gut microbiome is a very stable community of microbes composed of highly adapted microbial species [78,79]. The composition of the gut microbiome has been shown to be shaped more by environment than by host genetics [80]. Our analyses showed that senior orienteers as a group had a more homogenous microbiota, which makes individually stable microbiota profiles also more likely. This is not the case in the older adults’ samples. Individual stability over time has been observed as a feature that distinguishes the microbiota of healthy individuals compared with individuals with gastrointestinal disease [81]. Nevertheless, future studies need to validate our findings in a longitudinal study to verify that the gut microbiota is homogenous and stable among senior orienteers.

## 5. Conclusions

In conclusion, our data show that senior orienteers can be seen as a model of healthy ageing also from the perspective of the microbiota. Their faecal microbiota shows a higher abundance of Faecalibacterium prausnitzii that has been previously associated with positive health benefits, as well as an active lifestyle. In contrast, the senior orienteers have a lower abundance of Parasutterella excrementihominis and Bilophila wadsworthia, two species that previously have been associated with decreased intestinal health. Furthermore, our observation of senior orienteer faecal microbiota being more homogenous suggests this group of older adults as a model of healthy ageing.

## Figures and Tables

**Table 1:** Inclusion and exclusion criteria.

Older Adults	Senior Orienteering Athletes
Inclusion criteria	
Informed consent signed by the study participant Age ≥ 65 years Mentally and physically fit to complete questionnaires during the study period	Informed consent signed by the study participant Age ≥ 65 years Mentally and physically fit to complete questionnaires during the study period Actively performing and competing in orienteering
Exclusion criteria	
Any known gastrointestinal disease, malignancies, and ischemia Inflammatory bowel disease Participation in another clinical trial in the past three months	Any known gastrointestinal disease, malignancies, and ischemia Inflammatory bowel disease Participation in another clinical trial in the past three months

**Table 2:** Participant characteristics.

Parameter	Community-Dwelling Older Adults *n* = 70	Senior Orienteering Athletes *n* = 28	*p*-Value
Sex Median *n* (%)			
Female Male	33 (47%) 37 (53%)	12 (43%) 16 (57%)	0.701
Age Median (IQR)	72 (69–76)	68.5 (67–72)	0.034
Smoking *n* (%)	1 (1%)	0 (0%)	0.537
Physical activity Median (IQR)	3.5 (3–4)	4 (4–5)	<0.001 *
Polypharmacy *n* (%)	8 (12%)	2 (7%)	0.487
Number of medications Median (IQR)	2 (1–4)	1 (0–2)	0.016
GI symptoms Median (IQR)			
Indigestion Constipation Abdominal pain Diarrhoea Reflux	2.0 (1.3–3.1) 1.3 (1.0–3.3) 1.3 (1.0–2.0) 1.0 (1.0–3.3) 1.0 (1.0–1.5)	1.5 (1.3–1.9) 1.3 (1.0–1.6) 1.0 (1.0–1.7) 1.3 (1.0–1.7) 1.0 (1.0–1.0)	0.011 0.569 0.009 0.497 0.043
Total GI symptoms	1.8 (1.1–2.5)	1.3 (1.1–1.5)	0.021
Depression Median (IQR)	2 (1–4)	0 (0–1)	0.002 *
Anxiety Median (IQR)	2 (0.5–5.5)	0.5 (0–2.8)	0.006 *

* Retained significant difference after multiple testing corrections. Physical activity, GI symptoms, and psychological distress (depression and anxiety) are all measured with questionnaires, see the Materials and Methods section for a more detailed description of each questionnaire. Interquartile range (IQR) is presented within parentheses where applicable. GI = gastrointestinal.

## Supplementary Materials

The following are available online at https://www.mdpi.com/2072-6643/12/9/2610/s1, Figure S1: Work flow, Figure S2: Shannon_diversity, Table S1: Relative abundances: genera, species
