# Supplementary material for: Differences in Gut Microbiome Composition between Senior Orienteering Athletes and Community-Dwelling Older Adults
Source: Nutrients. 2020 Aug 27;12(9):2610. doi: 10.3390/nu12092610 (PMC7551621; doi:10.3390/nu12092610)
Supplement: Supplementary file 1 [file nutrients-12-02610-s001.zip › nutrients-887591-supplementary.pdf]

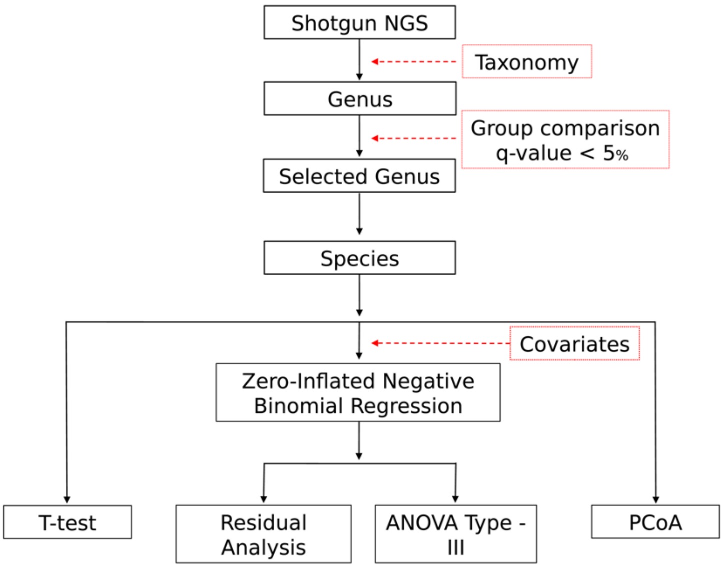

Figure S1. General workflow of microbiota analysis. Next generation shotgun sequences of microbiomes from faecal samples were taxonomically classified. Genera were selected based on false discovery rate (FDR), and species corresponding to these genera were selected for further analysis.

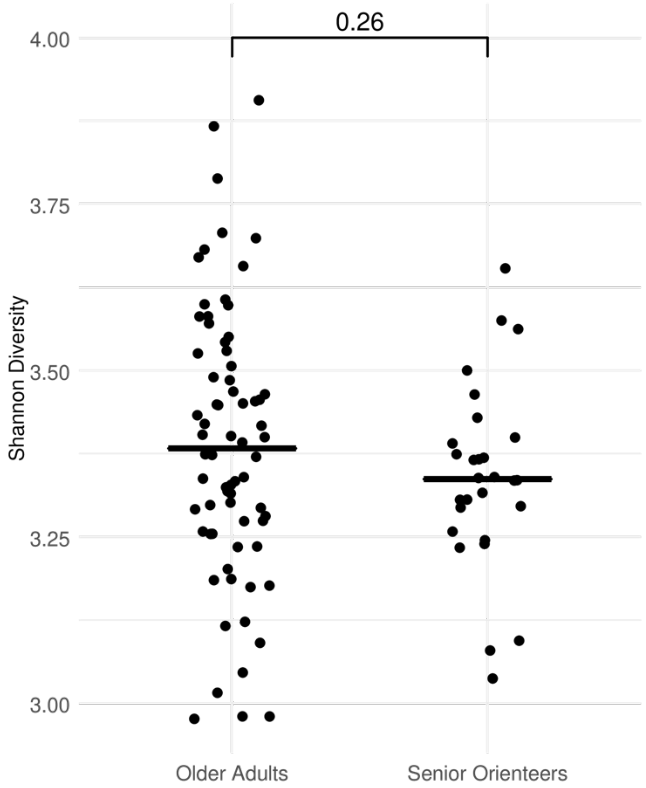

Figure S2. Species diversity among older adults and senior orienteers. Diversity scores for all predicted microbiomes were calculated using Shannon-Wiener diversity index and compared between older adults and senior orienteers
